# Supplementary material for: A recurrent point mutation in PRKCA is a hallmark of chordoid gliomas
Source: Nat Commun. 2018 Jun 18;9:2371. doi: 10.1038/s41467-018-04622-w (PMC6006150; doi:10.1038/s41467-018-04622-w)
Supplement: Supplementary file 1 — Supplementary Information [file 41467_2018_4622_MOESM1_ESM.pdf]

Supplementary Information File

A recurrent point mutation in *PRKCA* is a hallmark of Chordoid Gliomas

Rosenberg *et al.*

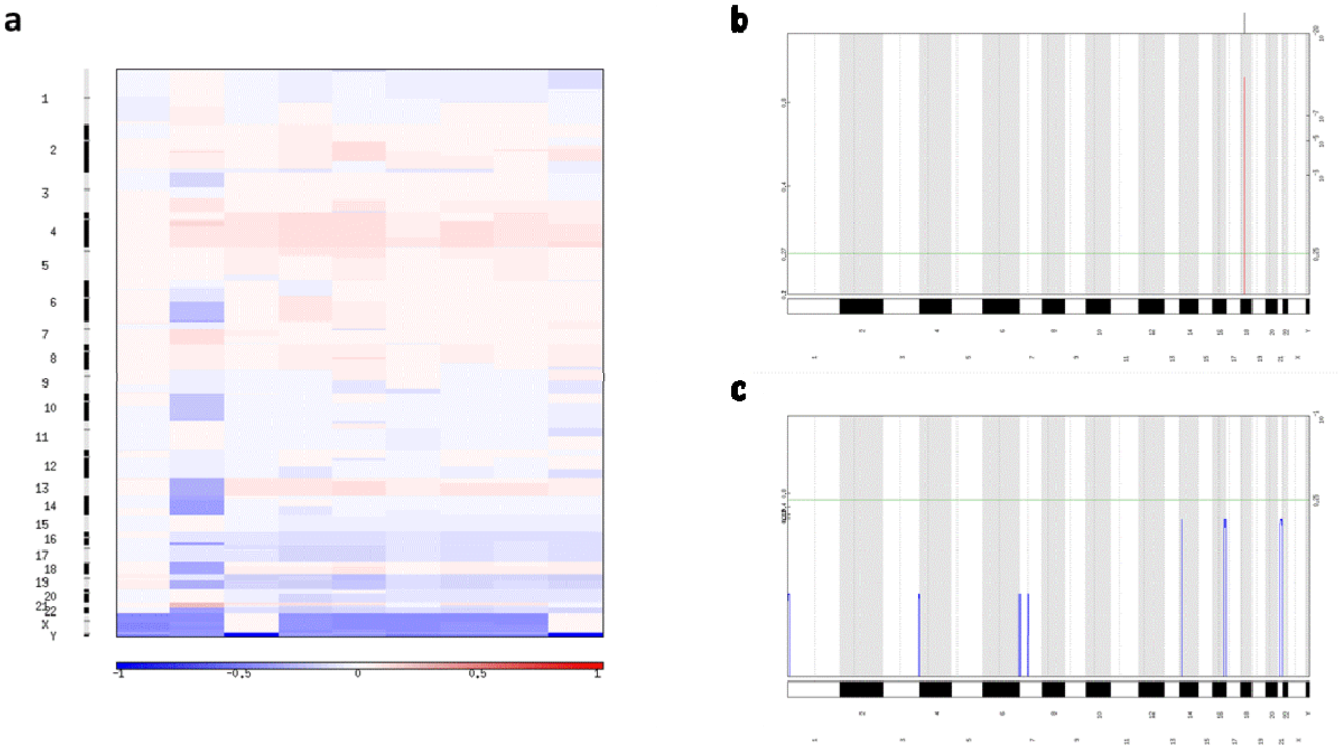

Supplementary Figure 1.a) chromosomal view: recurrent deletions of chromosomes 16, 17, 19, 22q and gain in 4q were noted. **b) focal amplifications:** 18q12.1 amplification which contains NOL4 and ASXL3 genes was found in 4/9 samples available. **c) focal deletions** did not pass the significant threshold.

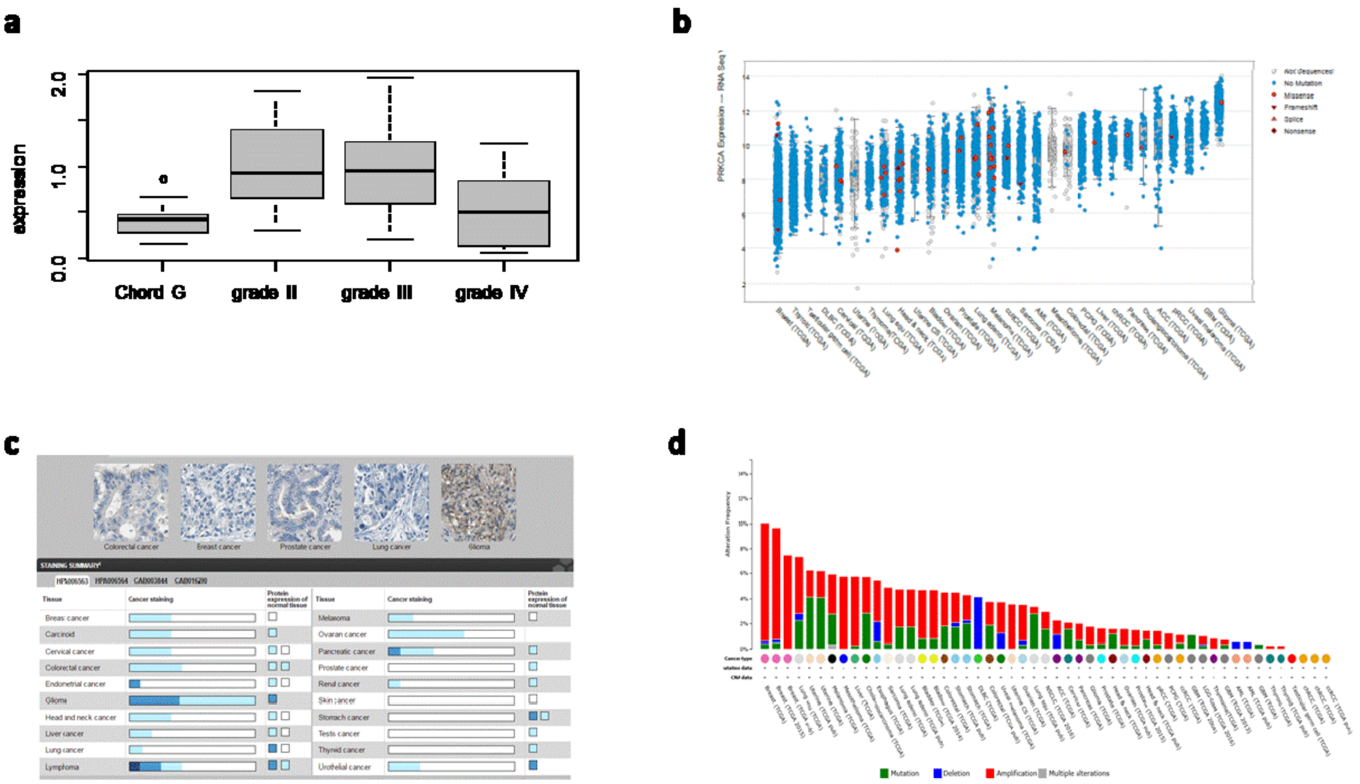

Supplementary Figure 2. Chordoid gliomas and Pan cancer PRKCa expression. RT-qPCR of PRKCA in our ChG and a panel of grade II to IV diffuse gliomas. *PPIA* (peptidylprolyl isomerase A (cyclophilin A)) was used as a control gene. Expression level, measured by $2^{\Delta CT(PRKCA\text{-}PPIA)}$, was similar for ChG and grade IV, and lower than grade II and III (a). Gliomas –and especially lower grade gliomas- have the highest expression of PRKCA expression both at the level of transcripts (b; boxplots with median and percentiles) and protein (c), despite a low prevalence of PRKCA mutations and amplifications compared to other tumors where PRKCA is frequently amplified or mutated (d).

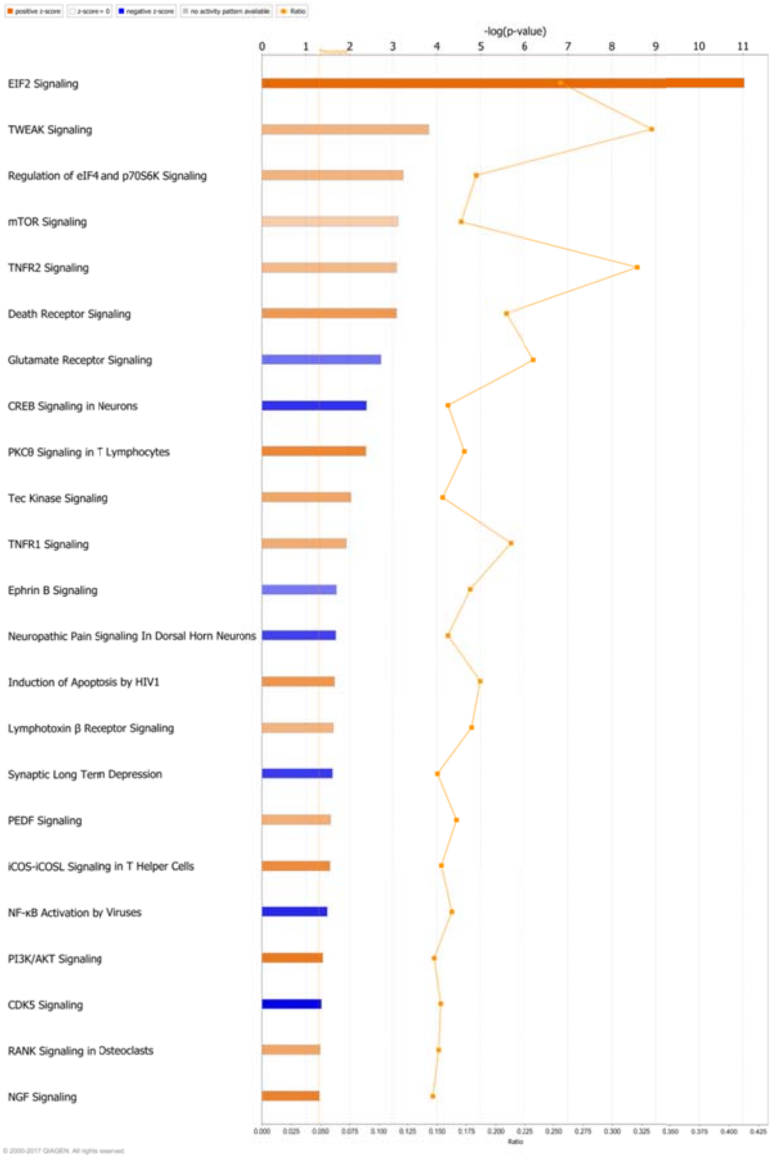

Supplementary Figure 3: Ingenuity Pathway Analysis (IPA) performed on differential expression analysis of G1+G2 ChG samples compared to TCGA grade II wild-type IDH. The barplot gives the p-value for each pathway - measured in logarithmic scale. Orange color represent pathway activation in ChG compared to grade II glioma and blue represent pathway inhibition. Higher color intensities represent stronger activation/inhibition of a pathway.

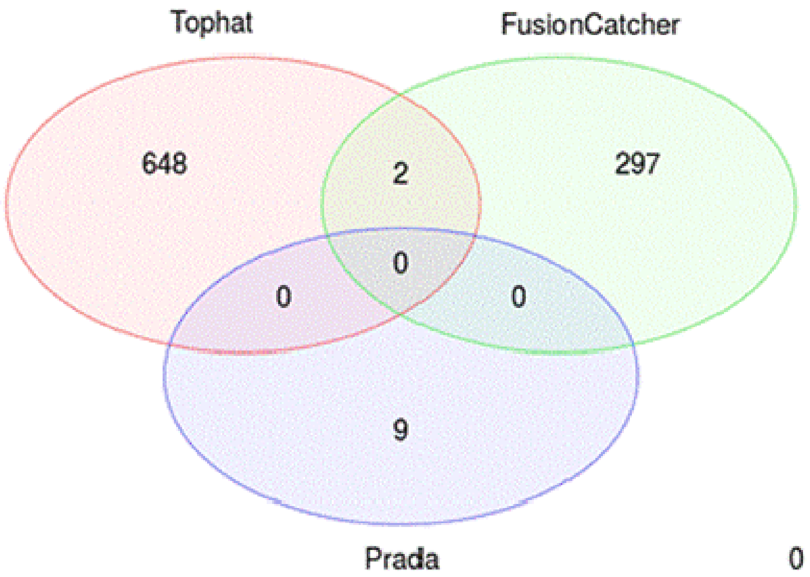

Supplementary Figure 4: Ven diagram of identified putative fusion transcripts. Fusion analysis used three different algorithms: (1) Tophat-fusion, (2) FusionCatcher, and (3) PRADA: the number of fusions after applying two filters (number of spanning pair reads=2, junction reads=1) is indicated.

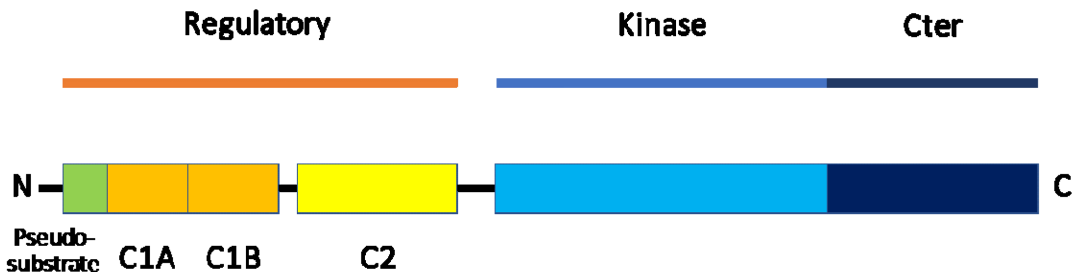

Supplementary Figure 5. General structure of conventional PRKCA (α, βI, βII,γ) (adapted from Newton, Am J Physiol Endocrinol Metab 298: E395–E402, 2010). PRKCA contains a pseudo-substrate domain that serves as an auto-inhibitor for the kinase domain function, C1A and C1B domains that bind Diacylglycerol and phorbol esters, a C2 domain, which binds Ca2+ and phosphatidylserine and the kinase domain, which phosphorylates PRKCA substrates and contains the D463H mutant residue.

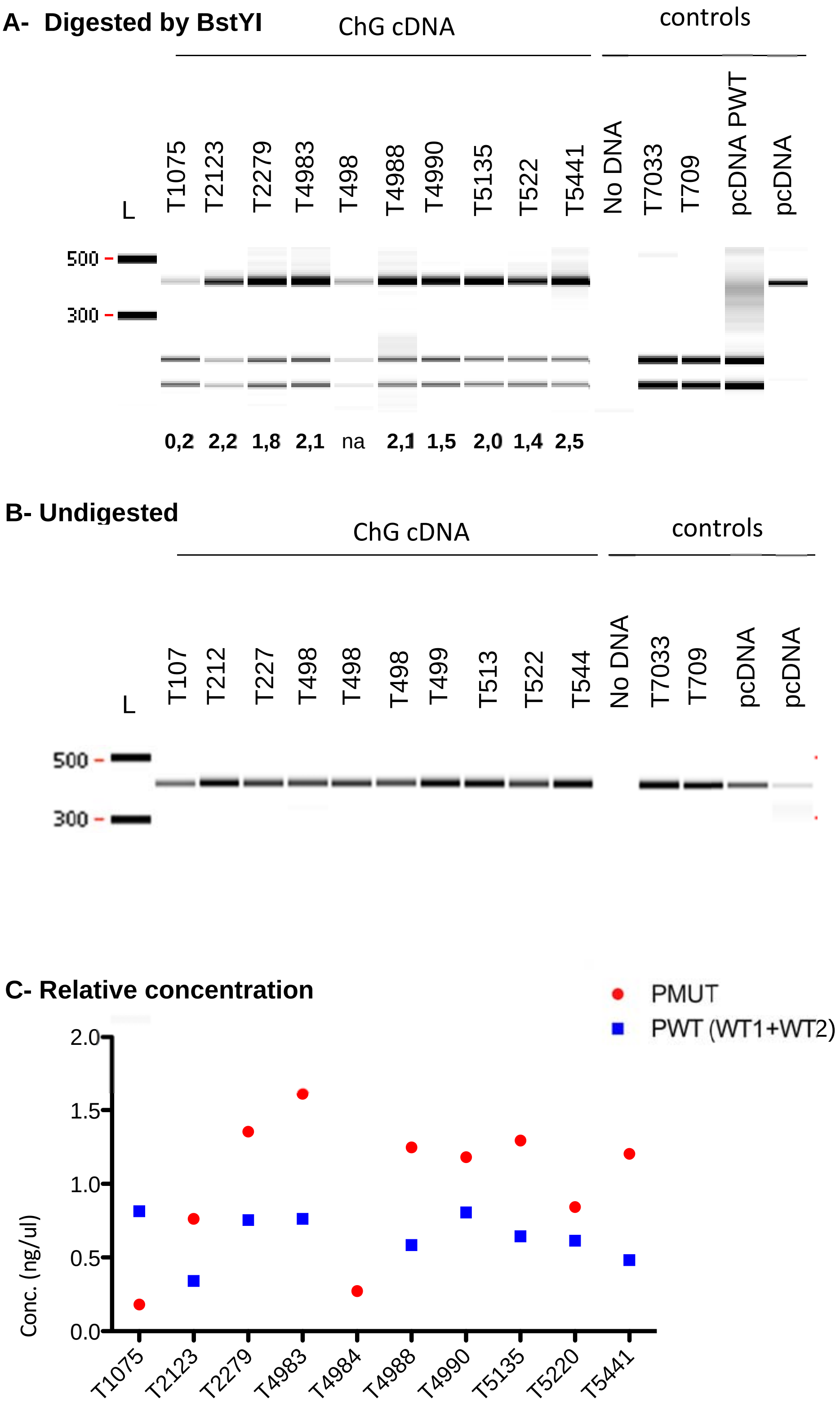
A- Digested by BstYI
ChG cDNA
controls
L
T1075
T2123
T2279
T4983
T498
T4988
T4990
T5135
T522
T5441
No DNA
T7033
T709
pcDNA PWT
pcDNA
500
300
0,2 2,2 1,8 2,1 na 2,1 1,5 2,0 1,4 2,5
B- Undigested
ChG cDNA
controls
L
T107
T212
T227
T498
T498
T498
T499
T513
T522
T544
No DNA
T7033
T709
pcDNA
pcDNA
500
300
C- Relative concentration
PMUT
PWT (WT1+WT2)
Conc. (ng/ul)
2.0
1.5
1.0
0.5
0.0
T1075
T2123
T2279
T4983
T4984
T4988
T4990
T5135
T5220
T5441

Supplementary Figure 6: Comparison of WT and mutant PRKCA cDNA abundance in chordoid glioma (ChG) samples heterozygos for the PRKCA mutation D463H. The G to C substitution leads to the loss of BstYI restriction site in the mutant allele. PCR products from ChG cDNA were either digested by BstYI (A), either left undigested (B), then analyzed by LabChIP GX. Lanes T7033 and T7095 correspond to cDNA from low grade glioma wild type for PRKCA, which shows that digestion efficiency is 100%. Mutant and undigested product size appears around 418 bp; wild type digested product sizes appears arround 227 bp and 185 bp. The last two lanes correspond to pcDNA plasmid controls, either WT or D463H mutant. (C) Diagram showing the concentrations (ng/ul) of $\mathit{PRKCA}^{\mathit{WT}}$(PWT) and $\mathit{PRKCA}^{\mathit{D463H}}$(PMUT).

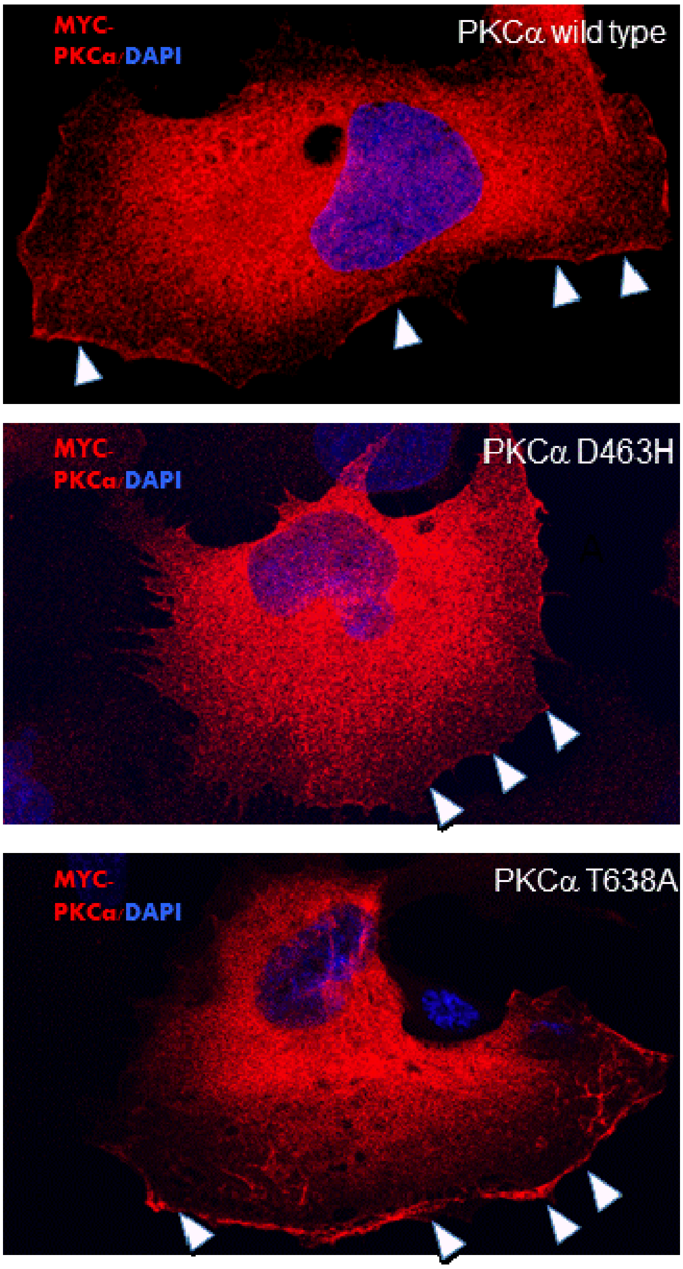

Supplementary Figure 7: IF anti-Myc shows a membrane labeling in human adult astrocytes transduced with *PRKCA*^{WT} and even more clear with the kinase dead mutant *PRKCA*^{T638A} (arrowheads). Membrane labeling is much weaker in *PRKCA*^{D463H} transfected astrocytes.

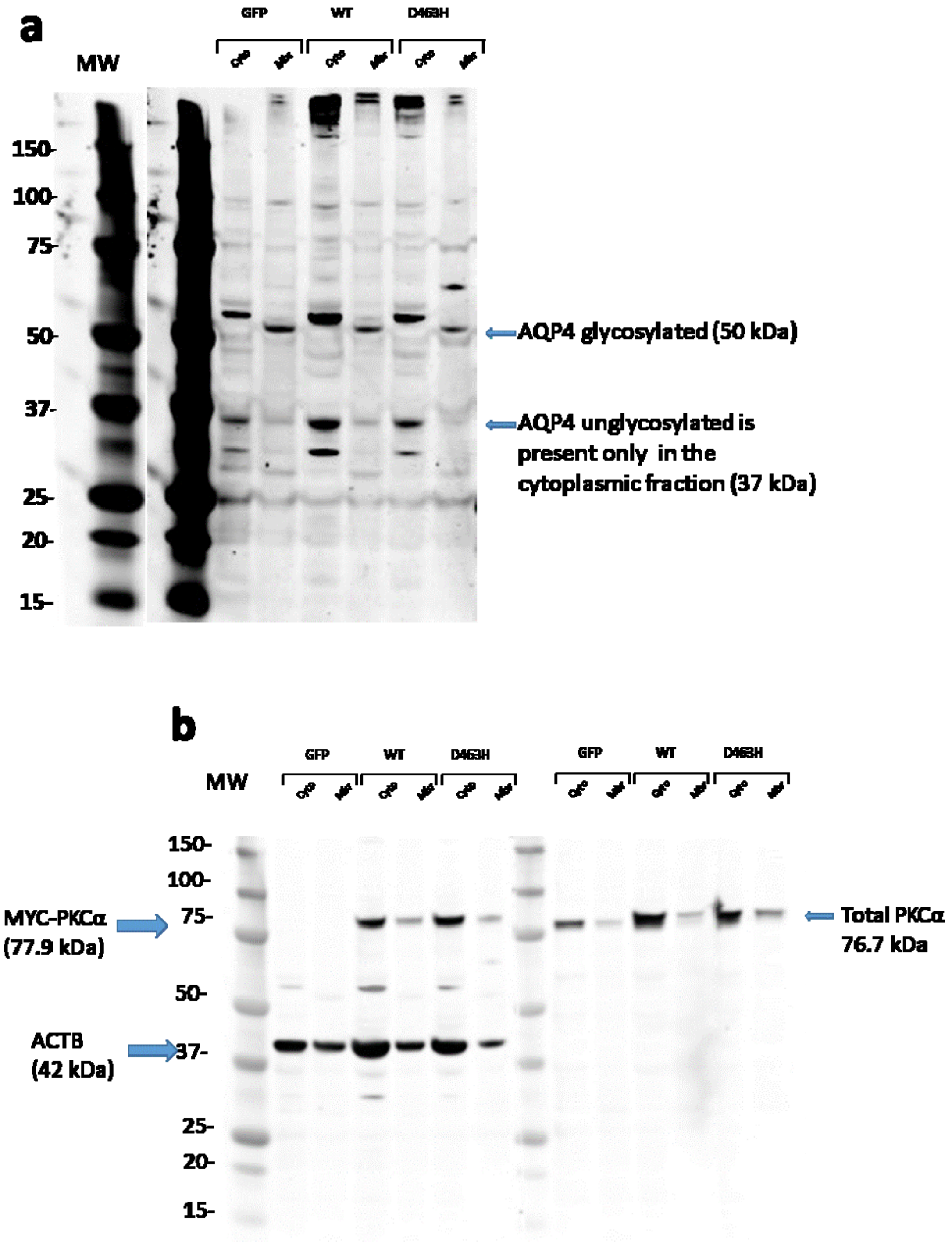
a
MW
GFP
WT
D463H
150-
100-
75-
50-
37-
25-
20-
15-
AQP4 glycosylated (50 kDa)
AQP4 unglycosylated is present only in the cytoplasmic fraction (37 kDa)
b
MW
GFP
WT
D463H
GFP
WT
D463H
150-
100-
75-
50-
37-
25-
20-
15-
MYC-PKCα (77.9 kDa)
ACTB (42 kDa)
Total PKCα 76.7 kDa

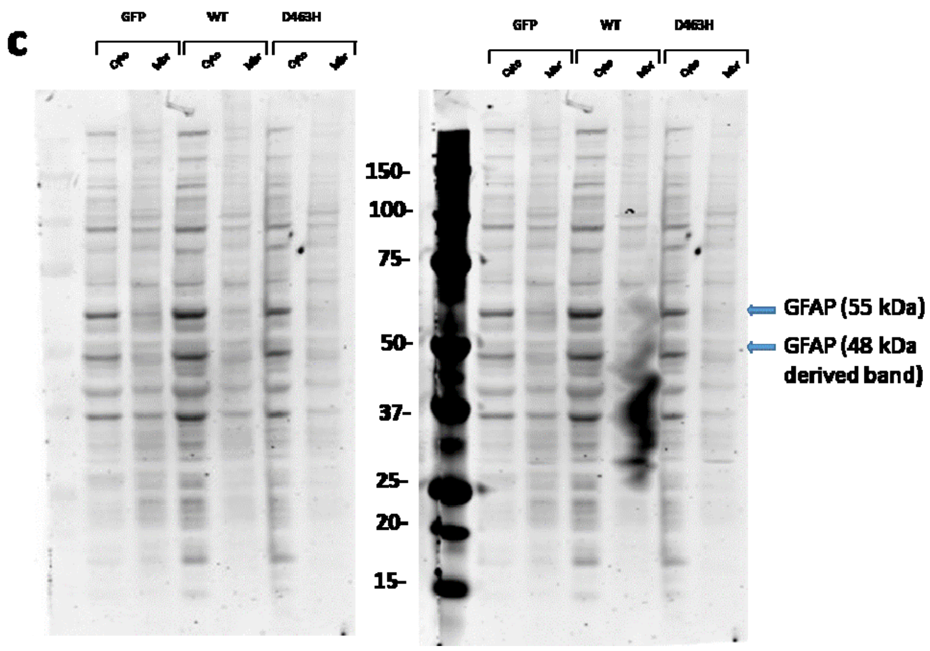

Supplementary figure 8: original autoradiographies of cellular fractionation: a) **AQP4, b) Total PKCα, Myc-PKCα and beta-actin c) GFAP**
