# Supplementary material for: A recurrent point mutation in PRKCA is a hallmark of chordoid gliomas
Source: Nat Commun. 2018 Jun 18;9:2371. doi: 10.1038/s41467-018-04622-w (PMC6006150; doi:10.1038/s41467-018-04622-w)
Supplement: Supplementary file 3 — Description of Additional Supplementary Files [file 41467_2018_4622_MOESM3_ESM.pdf]

Description of Additional Supplementary Files

File Name: Supplementary Data 1
Description: Recapitulate the mutational data on PRKCA and indicates the tumor purity.

File Name: Supplementary Data 2
Description: CNS tumor tested for PRKCAD463H tumor including rare tumors with similarities to chG, because of their juxta-ventricular location (ependymomas, neurocytomas, rosette-forming glioneuronal tumors of the fourth ventricle, choroid plexus tumors), or because they develop from circumventricular organs (papillary tumor of the pineal region, pituicytomas).

File Name: Supplementary Data 3
Description: The 346 somatic mutations identified in the four G1 samples: Somatic mutations in the four G1 tumor group after filtrations with SIFT/Polyphen prediction. Only PRKCAD463H (red) existed in all four samples and is predicted to have functional impact by Sift and Polyphen scores. Only two mutations existed in 2/4 samples: (i)PABPC3L530P which is not predicted to have a functional impact by Sift and Polyphen scores, and is not in a known cancer gene and (ii) chr6:26745595 which is not located in a gene (yellow). Out of 10 somatic mutations found among Cosmic gene census genes (see Figure 1C), only three of them were missense mutations with functional impact predicted by Sift or Polyphen score: FGFR4L661V, NCOR1P829Q and CDK12S293R (Green).

File Name: Supplementary Data 4
Description: GISTIC analysis for repeated chromosmal arm CNV events.

File Name: Supplementary Data 5
Description: GISTIC analysis for repeated focal CNV events.

File Name: Supplementary Data 6
Description: ssGSEA Pathways analysis by ssGSEA, based on groups G1 and G2. All pathways of Biocarta, PID and KEGG were analyzed.

File Name: Supplementary Data 7
Description: Ingenuity Pathway Analysis (IPA) on differential expression analysis of G1+G2 ChG samples compared to TCGA grade II IDH wild-type gliomas (Â© 2000-2017 QIAGEN. All rights reserved).

File Name: Supplementary Data 8
Description: EIF2 signaling pathway. Expression values are given if the gene was part of the top 2000 differentially expressed genes between ChG and TCGA grade II glioma IDH Wt

File Name: Supplementary Data 9
Description: Comparison of mutated PKCa to homologous proteins. PSI-Blastp alignment results for the kinase domain mutant sequence. Alignment is shown in the 5th column: mutant sequence is bold & underlined and the sequence below is the sequence of homologous protein. PSI-Blastp was carried out for the PKCa kinase domain sequence. There were 392 homologous proteins. We found 380 of them with aspartate at the position homologous for PRKCA 463, six proteins with histidine, and four proteins with other residues. Histidine can act as a proton acceptor or a donor of deprotonate substrate. Considering the different protonation states of histidine (Kim MO et al. J Comput Aided Mol Des. 2013 Mar;27(3):235-46), the occurrence of Histidine in a position analogous to PRKCA 463 in six homologous proteins, strengthens the hypothesis that the PRKCA D463H changes enzymatic activity or specificity in a gain of function manner.

File Name: Supplementary Data 10
Description: LabChip GX bioanalyzer row data, refering to the results shown in Figure 1d. BstYI restriction status is shown on the left. The Mut vs WT concentration ratio within each ChG tumor sample is calculated on the right, by sing the formula : Mut/WT ratio = Mut conc/(WT1 conc + WT2 conc), where WT1 and WT2 correspond to the two bands produced by the BstYI disgestion of the PCR amplicon. Note, that mutant cDNA is consistently enriched, compared to the WT cDNA, in all ChG but n°1. PWT is for PRKCA wild type. PMut is for PRKCA D462MH mutation. The last two lanes correspond to pcDNA plasmid controls, either WT or D463H mutant.

File Name: Supplementary Data 11
Description: Primers sequences.
